# Genome-wide identification and expression analysis of the HAK/KUP/KT gene family in Moso bamboo

**DOI:** 10.3389/fpls.2024.1331710

**Published:** 2024-03-26

**Authors:** Hui Guo, Jiaqi Tan, Yang Jiao, Bing Huang, Ruifang Ma, Muthusamy Ramakrishnan, Guoning Qi, Zhijun Zhang

**Affiliations:** ^1^ Bamboo Industry Institute, State Key Laboratory of Subtropical Silviculture, Zhejiang A&F University, Hangzhou, Zhejiang, China; ^2^ State Key Laboratory of Tree Genetics and Breeding, Co-Innovation Center for Sustainable Forestry in Southern China, Bamboo Research Institute, Key Laboratory of National Forestry and Grassland Administration on Subtropical Forest Biodiversity Conservation, College of Biology and the Environment, Nanjing Forestry University, Nanjing, Jiangsu, China

**Keywords:** bamboo, HAK, potassium ion transport, abiotic stress, gene expression, STEM

## Abstract

The K^+^ uptake permease/high-affinity K^+^/K^+^ transporter (KUP/HAK/KT) family is the most prominent group of potassium (K^+^) transporters, playing a key role in K^+^ uptake, transport, plant growth and development, and stress tolerance. However, the presence and functions of the KUP/HAK/KT family in Moso bamboo (*Phyllostachys edulis* (Carriere) J. Houzeau), the fastest-growing plant, have not been studied. In this study, we identified 41 *KUP/HAK/KT* genes (*PeHAKs*) distributed across 18 chromosomal scaffolds of the Moso bamboo genome. PeHAK is a typical membrane protein with a conserved structural domain and motifs. Phylogenetic tree analysis classified PeHAKs into four distinct clusters, while collinearity analysis revealed gene duplications resulting from purifying selection, including both tandem and segmental duplications. Enrichment analysis of promoter cis-acting elements suggested their plausible role in abiotic stress response and hormone induction. Transcriptomic data and STEM analyses indicated that *PeHAKs* were involved in tissue and organ development, rapid growth, and responded to different abiotic stress conditions. Subcellular localization analysis demonstrated that *PeHAKs* are predominantly expressed at the cell membrane. *In-situ* PCR experiments confirmed that *PeHAK* was mainly expressed in the lateral root primordia. Furthermore, the involvement of *PeHAKs* in potassium ion transport was confirmed by studying the potassium ion transport properties of a yeast mutant. Additionally, through homology modeling, we revealed the structural properties of HAK as a transmembrane protein associated with potassium ion transport. This research provides a solid basis for understanding the classification, characterization, and functional analysis of the PeHAK family in Moso bamboo.

## Introduction

Potassium (K^+^) is the second most crucial macronutrient for plant growth, following nitrogen (N), and contributes up to 10% of the total plant biomass (Ahammed et al., 2022). K^+^ transportation in plants primarily occurs through potassium transporter and ion channel protein families (Castillo et al., 2015; Li et al., 2018). Among these transporter protein families, the KUP/HAK/KT family (abbreviated as HAK) is the largest and plays a crucial role in mediating intracellular K^+^ accumulation for maintaining plant growth and development (Ahn et al., 2004; Véry et al., 2014). HAK operates as a K^+^/H^+^ symporter, enhancing K^+^ uptake in plants by coupling high-affinity potassium translocation with H^+^ currents (Santa-María et al., 2018; Sze and Chanroj, 2018) and typically consist of 10-14 transmembrane domains (TM) with a long loop in the second to third transmembrane region. Both the C- and N-terminal ends of HAK proteins are located inside the cell, with the C-terminus being longer than the N-terminus. These terminal ends are critical for ion recognition, binding, and regulating the rate of potassium ion transport and ion homeostasis inside and outside the cell (Li et al., 2018; Santa-María et al., 2018).

*HAK* are responsible for K^+^ uptake and transport in various plant species (Mäser et al., 2001; Yang et al., 2009; Zhang et al., 2012; Cai et al., 2021)and mediate K^+^ translocation in different tissues (Martinez-Cordero et al., 2004; Boscari et al., 2009; Yang et al., 2014; Chen et al., 2015, 2019). For example, in Arabidopsis, *AtHAK5* and *AtKT1* are the primary members and responsible for K^+^ uptake in the root system. *AtHAK5* is involved in high-affinity K^+^ uptake, especially in external low potassium (<10 µM), and it maintains high expression levels even after one week of K^+^ starvation treatment (Gierth et al., 2005; Rubio et al., 2008; Ragel et al., 2015), suggesting that *AtHAK5* promotes K^+^ uptake by Arabidopsis roots. On the other hand, *AtKT1* mediates K^+^ uptake under low K^+^ conditions (Quintero and Blatt, 1997). *OsHAK1* is pivotal in mediating K^+^ uptake and translocation across both K^+^ uptake systems, accounting for approximately 50-55% of K^+^ uptake at external K^+^ concentrations of 0.05-0.1 mM, and about 30% at 1 mM (Chen et al., 2015). *OsHAK5*, prominently expressed in the root epidermis, mesocotyls, and vascular tissues, is integral not only to K^+^ acquisition but also to the transfer of K^+^ from roots to shoots, especially under conditions of low external K^+^ (Yang et al., 2014). In barley, *HvHAK4* is involved in K^+^ uptake and translocation to leaves, where it ensures a higher concentration of chloroplastic K^+^ compared to that in the epidermis (Boscari et al., 2009; Cuin et al., 2011). A recent study has shown that rice phloem K^+^ loading and transport is dependent on *OsHAK18*, which mediates both potassium and sodium cycling and sugar transport in rice (Peng et al., 2023).

The sensing of changes in external or internal K^+^ concentrations is key to regulating K^+^ homeostatic balance (Adams and Shin, 2014). In the K^+^ starvation signaling pathway, reactive oxygen species (ROS), Ca2^+^, and phytohormones are known regulatory signals (Shin and Schachtman, 2004; Amtmann and Armengaud, 2007; Li et al., 2018). In Arabidopsis, *AtHAK5* expression is modulated by ethylene signaling, which enhances ROS production, leading to increased *AtHAK5* expression and K^+^ accumulation under low-potassium conditions, thus elevating the K^+^/Na^+^ ratio (Jung et al., 2009; Kim et al., 2010). Furthermore, ROS production is regulated by various oxidative enzymes and peroxidases. For instance, the peroxidase RCI3 (rare cold inducible gene 3) regulates ROS production in the absence of K^+^, affecting *AtHAK5* expression (Kim et al., 2010). Further studies have revealed that the GCC-box site in the *AtHAK5* promoter binds to the transcription factor AP2/ERF (Kim et al., 2012). This factor’s expression is influenced by ROS, ethylene, and low potassium levels, playing a role in root growth and K^+^ uptake (Kim et al., 2012; Li et al., 2018). Similar to the inwardly rectifying potassium channel AKT1, *AtHAK5* is co-regulated by the calcium-regulated signal CBL (Ca2^+^ sensors calcineurin B-like) and the protein kinase CIPK (CBL-interacting protein kinase), which are involved in high-affinity K^+^ uptake in Arabidopsis roots (Ragel et al., 2015; Scherzer et al., 2015). Additionally, the C-terminus of AtKUP6 is influenced by ABA (abscisic acid) signaling. It is phosphorylated by SRK2E (SNF1-related protein kinases 2E), a protein kinase from the PYR (pyrabactin resistance) family of ABA signaling receptors, playing a role in ABA-mediated stomatal closure under water stress (Osakabe et al., 2013). This intricate network of signaling pathways and molecular interactions underscores the complexity of maintaining K^+^ uptake and transport in plants.

In addition to their involvement in K^+^ uptake, transport, and translocation, stress-induced *HAK* expression also regulates stress tolerance, and plant growth and development (Li et al., 2018; Huang et al., 2019; Yang et al., 2020; Ankit et al., 2022). Despite the inhibitory effect of high Na^+^ concentrations on *HAK* expression (Nieves-Cordones et al., 2008), *AtHAK5* in Arabidopsis can still uptake K^+^ even at high Na^+^ concentrations (Gobert et al., 2006; Rubio et al., 2008). Other *HAK* members, such as *AtKUP6*, *AtKUP11*, *SlHAK20*, *OsHAK1*, *OsHAK5*, and *OsHAK21* (Gobert et al., 2006; Rubio et al., 2008; Wang et al., 2020), play a crucial role in maintaining K^+^/Na^+^ homeostasis and enhancing stress tolerance in plants (Yang et al., 2014; Chen et al., 2015; Shen et al., 2015; Wang et al., 2020). AtKUP4 is essential for correctly positioning the PIN1 protein at the root tip, with mutations in AtKUP4 disrupting growth hormone distribution and efflux rates (Rigas et al., 2013; Daras et al., 2015). It is believed that AtKUP4 acts as a vital link between root hair development and environmental/hormonal signaling, crucial for maintaining growth hormone balance during environmental adaptation in plants (Ahn et al., 2004; Rigas et al., 2013; Daras et al., 2015; Li et al., 2018). Additionally, mutations in *OsHAK1*, *OsHAK5*, *OsHAK16*, *AtHAK5*, and *SiHAK1* have been observed to adversely affect root development, characterized by delayed root growth, growth inhibition, and reduced germination rates (Yang et al., 2014; Zhang et al., 2018; Feng et al., 2019). Although the essential functions of *HAK* genes have been characterized in several plant species, the mechanisms governing *HAK* regulation remain poorly understood.

Moso bamboo, a large and rapidly-growing woody bamboo species, is widely distributed in East and Southeast Asia (Song et al., 2020). This species holds significant cultural, ecological value, industrial, and economic value (Pan et al., 2017; Chen et al., 2022). Due to its high productivity, strength, and abundance of resources, Moso bamboo finds extensive applications in renewable energy, construction, food, and medicine (Ramakrishnan et al., 2020). However, the HAK gene family of Moso bamboo remains unexplored.

Considering the vital functions of *HAK* in plant growth and development, this study focused on identifying putative *PeHAKs* at the genome-wide level and analyzed their chromosomal locations, phylogenetic relationships, gene structures, conserved domains, motifs, *cis*-elements, and synteny. Additionally, we investigated the subcellular and tissue localization of these genes, performed protein homology modeling, and explored the potential K^+^ pore section. Furthermore, we examined the expression profiles of *PeHAKs* in different tissues, under various plant hormone treatments, and in response to diverse abiotic stresses. Notably, our findings suggested the direct involvement of *HAK* in rapid growth and shoot development. This work provides valuable insights into *PeHAK* gene functions related to tissue growth and organ development in Moso bamboo.

## Materials and methods

### Identification and physicochemical properties of the *HAK* gene family

The genome files for the Moso bamboo genome were downloaded from public databases. The Hidden Markov Model (HMM) of the K-Trans protein signature structural domain (PF02705) of the HAK gene family was downloaded from the Pfam database (http://pfam.xfam.org/). The local-protein database (E ≤ 10^-20^) was searched using HMMER3 (http://hmmer.org/) (Finn et al., 2011), and the protein structural domains were validated using CCD (https://www.ncbi.nlm.nih.gov/cdd) (Finn et al., 2016).

Additionally, transmembrane structures were predicted using TMHMM-2.0 (https://services.healthtech.dtu.dk/services/TMHMM-2.0/). Genes that did not exhibit the characteristic features of the HAK family, primarily due to the presence of atypical or residual transmembrane structural domains, were excluded. This process resulted in the identification of candidate gene families more representative of typical HAK family characteristics (Cai et al., 2021). The basic information of the candidate gene family, such as protein length, molecular weight, isoelectric point, and instability coefficient, was predicted by ProtParam(https://web.expasy.org/protparam/), and the signal peptide and subcellular localization were predicted using SignalP 4.1 online software server and WoLF PSORT(https://www.genscript.com/tools/wolf-psort), respectively (Gasteiger et al., 2005).

### Phylogenetic analysis of the *HAK* gene family

Twenty-seven amino acid sequences of HAK from rice and 13 amino acid sequences of HAK from Arabidopsis were obtained from the rice genome database RGAP (http://rice.plantbiology.msu.edu/) and the Arabidopsis database TAIR (https://www.arabidopsis.org/), respectively. These sequences were aligned with Moso bamboo sequences using ClustalX2.1 software, and the sequence alignment results were used to construct a phylogenetic tree using MEGA11 software with the Maximum likelihood (ML) method and 1000 bootstrap replicates for evaluation.

### Analysis of conserved structural domains, motifs, and gene structures

The conserved structural domains and motifs of HAK family members were predicted using online resources, including NCBI (Marchler-Bauer et al., 2005) and MEME (Bailey et al., 2006). Gene structures were created using TBtool software by annotating the moso bamboo genome files downloaded from GigaDB (https://ngdc.cncb.ac.cn/databasecommons/database/id/4151) (Ma et al., 2018; Huang et al., 2021).

### Analysis of cis-acting elements in *PeHAK* promoters

The promoter sequence of each *PeHAK* gene, consisting of a 1500 bp upstream nucleotide sequence, was retrieved. To identify *cis*-acting elements within the promoter regions, the PlantCARE online database (https://bioinformatics.psb.ugent.be/webtools/plantcare/html/) was used. The results were sorted, enriched through screening, and visualized accordingly (Yang et al., 2020; Cai et al., 2021).

### Chromosome distribution, collinearity analysis, and Ka/Ks ratio

The positional information and chromosome lengths of *HAK* gene members from *P. edulis*, *O. sativa*, *Z. mays*, and *B. distachyon* were obtained from MG2Cv.2 (http://mg2c.iask.in/MG2C_v2.0/) (Chao et al., 2015; Yang et al., 2023). These data were compared and visualized through covariance analysis using the Multiple Co-linear Scanning Toolkit (MCScanX) (Wang et al., 2012). In Moso bamboo, 17 homologous gene pairs were identified through intraspecific covariance BLAST. Subsequently, the identified gene pairs underwent Ka/Ks analysis. To calculate synonymous substitution rates (Ks), non-synonymous substitution rates, and Ka/Ks ratios for the *HAK* genes, the Moso bamboo-specific differentiation time equation T = Ks/2λ was employed, where λ represents 6.5 × 10^-9, the estimated evolutionary differentiation time for Moso bamboo (Li et al., 1987; Peng et al., 2013). The KaKs_Calculator2 tool was used for the computation of these rates and ratios (Chen et al., 2023).

### Expression analysis of *PeHAK* genes

Gene expression datasets of roots, rhizomes, panicles, and leaves of Moso bamboo were acquired from the EMBL database (PRJEB2956). Transcriptome data of the shoot tissues at 0.5, 1, 2, 3, 5, 6, and 7 meters were downloaded from the NCBI-SRA database (PRJNA414226). Additionally, transcriptome data of the seedling root tissues treated with 5 μM gibberellin (GA) and 5 μM naphthalene acetic acid (NAA) were obtained from the NCBI-SRA database (PRJNA413166). These datasets were used to calculate the expression abundance of *PeHAKs* separately, measured as transcripts per million reads (TPM) values (**Supplementary Table 3**). For statistical convenience, each expression TPM value was log2 ^(TPM+1)^ transformed, and TBtools was used to create a gene expression heatmap (Lama et al., 2020). Furthermore, trend analysis of *PeHAKs* gene expression during the rapid growth of Moso bamboo shoots was performed using the STEM (Short Time-series Expression Miner) clustering method (Ernst and Bar-Joseph, 2006).

### Plant material, RNA extraction, and qRT-PCR analysis of genes

Moso bamboo seeds were harvested from Guilin, Guangxi, China, and seedlings were cultured in a greenhouse for one month with a 16 h light/8 h dark photoperiod and an average temperature of 22°C.One-month-old healthy Moso bamboo seedlings were selected to analyze the expression pattern of *PeHAK* genes under abiotic stress treatments. To mimic drought and salt stress conditions, we introduced 30% PEG6000 and 200 mM NaCl into the hydroponic nutrient solution for Moso bamboo, respectively (Zhang et al., 2022). Additionally, for high and low-temperature treatments, seedlings were placed in a light incubator at 42°C and 4°C for 0 h, 3 h, 6 h, 12 h, and 24 h, respectively.

Three biological replicates were collected for each treatment and control. The samples were immediately frozen in liquid nitrogen and stored at -80°C for further analysis. Total RNA was extracted from each sample using the FastPure Plant Total RNA Extraction Kit (Vazyme Company, China). The first-strand cDNA was synthesized using the HiScript^®^ III 1st Strand cDNA Synthesis Kit (+gDNA wiper) (Vazyme Company, China), where the gDNA wiper was utilized to eliminate DNA contamination. Specific primers were designed using Beacon Designer 7.0, and all primer sequences are listed in **Supplementary Table 2**. We selected *PeNTB*, a gene exhibiting stable expression across various Moso bamboo tissues, as our preferred internal reference gene for the study (Fan et al., 2013), and qRT-PCR analysis was performed on three replicates of each sample using a CFX-96 Real-Time system (Bio-Rad, United States). Relative gene expression was calculated using the 2^(-ΔΔCT)^ method (Livak and Schmittgen, 2001) and expressed as the mean ± standard deviation (SD). The significance of differences was assessed using ANOVA (one-way analysis of variance) and visualized using GraphPad Prism 7.

### Gene ontology enrichment analysis

Gene ontology (GO) is used to fully characterize the properties and products of genes in an organism. GO encompasses three different ontologies, describing the molecular function, cellular component, and biological process of genes (Harris et al., 2004). To investigate the involvement of *PeHAKs* in biological processes, GO enrichment prediction was performed using online software (https://cloud.majorbio.com/) (Thomson et al., 2019).

### Subcellular and tissue localization analysis

The subcellular localization of *PeHAK* members was predicted to be at the cell membrane. To validate this localization, the full-length CDS sequence was amplified using a seamless PCR cloning technique. The resulting fragment was then ligated into the pCAMBIA1300-35S-GFP vector containing the green fluorescent protein (EGFP) reporter gene and driven by the 35S promoter, using NovoRec® plus One step PCR Cloning Kit(No:NR005) (Novoprotein, Shanghai, China). After successful cloning, the recombinant vector was transformed into *Agrobacterium tumefaciens* GV3101 and injected into 5-week-old *Nicotiana benthamiana* leaves (Ma et al., 2021). The green fluorescence was observed using a confocal laser microscope (Olympus, Tokyo, Japan).

For tissue localization analysis, root tip sections of one-month-old Moso bamboo seedlings were collected. The sections were embedded and fixed in 5% agarose and then cut into 50-μm-thick sections using a microtome. The tissue sections were collected in 200μL tubes and subjected to DNase I enzyme treatment (**37**°C, 45min). Afterward, reverse transcription and PCR were carried out using an *in-situ* PCR system, and the resulting cDNAs were amplified with gene-specific primers (**Supplementary Table 4**). Subsequently, the samples were washed and incubated with alkaline phosphatase-conjugated anti-digoxin Fab fragments. Color development using the BM purple AP substrate was performed in the dark. Positive controls were conducted using *PeACT*, while negative controls omitted the reverse transcription step (RT).

### Validation of potassium ion transport properties

To validate the potassium transport activity of HAK, we selected the highly expressed *PeHAK37* and the lowly expressed *PeHAK04* genes in abiotic stress experiments for yeast-deficient complementation experiments. The complete coding sequences of *PeHAK04* and *PeHAK37* were amplified by PCR, and the P416 expression vector was successfully constructed and ligated through double digestion (**Supplementary Table 4**). The PeHAK04-P416 and PeHAK37-P416 plasmids were extracted and transformed into the K^+^ uptake-deficient strain R5421 of *Saccharomyces cerevisiae* (Zhang et al., 2020). Positive clones were selected along with yeast transformants carrying the empty vector and recombinant plasmid, based on consistent growth. These transformants were then incubated overnight in -Ura liquid medium at 28°C and 200rpm until they reached an OD600 value of 0.5. Subsequently, they were incubated on different solid AP media with varying concentrations of K^+^ (1 mM, 10 mM, and 100 mM) at both 28°C and a high temperature of 37°C. Similarly, they were also incubated on AP medium (5 mM) with different concentrations of Na^+^ (10 mM, 50 mM, 250 mM, 500 mM) at 28°C. Colony growth was observed, and photographs were taken (Liu et al., 2019).

### Protein tertiary structure analysis

HAK proteins are integral in facilitating the transmembrane transport of substances. Within this protein family, *PeHAK25* stands out as a potential candidate due to its characteristic transmembrane structure. To further characterize its properties, we selected *PeHAK25* for comprehensive analyses, including tertiary structure modeling and functional prediction. To predict and analyze the protein’s surface hydrophilicity and electrostatic potential distribution, Discovery Studio (https://www.3ds.com/) was employed. Additionally, PoreWalker software (https://www.ebi.ac.uk/thornton-srv/software/PoreWalker/) (Pellegrini-Calace et al., 2009) was utilized to predict the pore morphology.

## Results

### Identification of the *HAK* gene family

The HMM was used to search for potential family members, resulting in the identification of 55 candidates. The subsequent screening revealed the presence of 41 PeHAK family members (**Table 1**). Gene names ranging from *PeHAK1* to *PeHAK41* were assigned based on their chromosomal position. The corresponding amino acid sequences were subjected to bioinformatics analysis, including an assessment of their physicochemical properties. The results showed that *PeHAK* lengths ranged from 390 to 1252 amino acids (aa). The largest protein had a molecular weight of 137.99 kD, while the smallest protein weighed 58.22 kD, with an average molecular weight of 85.95 kD. The lengths of the amino acid sequences ranged from 83 to 403 aa. The isoelectric points of the proteins fell within the range of 6.95 to 9.13. Among the family members, 39 proteins were classified as basic (theoretical isoelectric points > 7), while 2 were considered acidic (theoretical isoelectric points < 7). The analysis indicated that the majority of Moso bamboo HAK family members encoded basic proteins.

**Table 1:** Information on *HAK* family genes in the moso bamboo.

Gene name	Gene ID	Chromosomal locus	MW (kDa)	PI	AI	GRAVY	SP	TMS	PL
*PeHAK01*	*PH02Gene16473.t1*	s3:90593551:90599713:+	85.73	8.21	106.65	0.341	No	13	Va
*PeHAK02*	*PH02Gene36140.t1*	s3:2050573:2066804:+	88.03	8.7	108.5	0.363	No	13	Va
*PeHAK03*	*PH02Gene37387.t1*	s3:1831590:1834426:+	58.22	8.66	108.85	0.397	No	9	CM. Va.
*PeHAK04*	*PH02Gene17972.t1*	s4:2129298:2133056:+	87.23	7.6	110.13	0.34	No	11	Va
*PeHAK05*	*PH02Gene21781.t1*	s4:2943291:2957241:+	87.10	8.88	108.18	0.422	No	12	Va
*PeHAK06*	*PH02Gene47873.t1*	s4:59157646:59163271:+	97.42	8.87	103.44	0.243	No	11	Va
*PeHAK07*	*PH02Gene05312.t1*	s6:22303148:22308774:-	84.93	8.32	108.39	0.357	No	12	Va
*PeHAK08*	*PH02Gene34335.t1*	s6:31283470:31295353:-	83.01	8.93	108.24	0.41	No	12	CM. Va
*PeHAK09*	*PH02Gene40368.t1*	s6:38250843:38255664:+	89.29	8.55	104.58	0.321	No	11	Va
*PeHAK10*	*PH02Gene17438.t1*	s8:72047466:72069902:+	93.12	8.84	105.26	0.299	No	12	CM. Va
*PeHAK11*	*PH02Gene32783.t1*	s8:27305838:27310995:-	84.93	8.03	109.4	0.366	No	12	Va
*PeHAK12*	*PH02Gene43347.t1*	s8:62953070:62958858:-	137.99	8.65	98.45	0.235	No	10	Va
*PeHAK13*	*PH02Gene03090.t1*	s9:23799715:23801479:-	43.33	9.1	122.38	0.781	No	9	Va
*PeHAK14*	*PH02Gene47801.t1*	s10:55908562:55916991:-	98.11	8.78	106.49	0.288	No	12	Va
*PeHAK15*	*PH02Gene16598.t1*	s13:46032872:46037359:+	88.50	8.32	108.78	0.39	No	14	Va
*PeHAK16*	*PH02Gene37003.t1*	s13:60323255:60325485:-	71.96	8.78	111.3	0.43	No	10	CM. Va
*PeHAK17*	*PH02Gene37200.t1*	s13:53227805:53234548:-	82.38	8.4	107.63	0.395	No	12	CM. Va
*PeHAK18*	*PH02Gene47464.t1*	s13:60460057:60463029:-	79.07	9.05	109.47	0.405	Yes	9	CM. Va
*PeHAK19*	*PH02Gene09194.t1*	s14:83247886:83254170:+	87.51	9.13	108.53	0.414	No	13	CM. Va
*PeHAK20*	*PH02Gene14233.t1*	s14:82647983:82650945:-	76.76	8.55	101.67	0.393	No	10	CM. Va
*PeHAK21*	*PH02Gene28171.t1*	s14:15526314:15530403:-	81.21	7.64	104.73	0.389	No	11	Va
*PeHAK22*	*PH02Gene26505.t1*	s15:44319072:44325299:-	87.85	6.73	108.27	0.335	No	11	CM. Va
*PeHAK23*	*PH02Gene40424.t1*	s15:20355020:20369994:+	90.10	8.87	108.65	0.302	No	11	CM. Va
*PeHAK24*	*PH02Gene47195.t1*	s15:19165715:19170114:+	90.87	8.78	108.86	0.305	No	11	Va
*PeHAK25*	*PH02Gene12083.t2*	s16:5992929:5998273:-	87.22	8.84	106.69	0.297	No	12	CM. Va
*PeHAK26*	*PH02Gene08121.t2*	s17:14661448:14667154:-	85.68	8.35	108.91	0.383	No	13	Va
*PeHAK27*	*PH02Gene16414.t1*	s18:39130195:39152028:-	79.26	8.98	106.6	0.381	No	11	CM. Va
*PeHAK28*	*PH02Gene17639.t1*	s19:23944880:23950239:-	87.10	8.57	107.43	0.404	No	13	Va
*PeHAK29*	*PH02Gene19068.t1*	s19:26122442:26126331:-	87.09	8.34	111.88	0.352	No	11	Va
*PeHAK30*	*PH02Gene48788.t1*	s20:2051844:2055146:-	83.30	8.57	103.59	0.339	No	12	CM. Va
*PeHAK31*	*PH02Gene12427.t1*	s21:69969721:69976204:-	87.31	6.95	109.12	0.365	No	12	Va
*PeHAK32*	*PH02Gene40148.t1*	s21:44584712:44589587:+	94.33	9.04	110.58	0.332	No	12	CM. Va
*PeHAK33*	*PH02Gene41444.t1*	s21:44224269:44230516:-	90.14	8.49	111.78	0.313	No	10	CM. Va
*PeHAK34*	*PH02Gene41445.t1*	s21:44072677:44077215:-	83.92	8.76	109.15	0.385	No	11	CM. Va
*PeHAK35*	*PH02Gene15453.t1*	s22:8538417:8545286:-	82.63	8.19	106.57	0.399	No	12	CM. Va
*PeHAK36*	*PH02Gene01748.t4*	s23:11534415:11538274:+	82.90	9.03	117.03	0.56	No	12	CM. Va
*PeHAK37*	*PH02Gene01771.t1*	s23:11000240:11008737:+	88.39	7.83	111	0.379	No	13	CM. Va
*PeHAK38*	*PH02Gene29364.t1*	s23:43326384:43329523:-	86.73	9.09	105.99	0.271	No	13	Va
*PeHAK39*	*PH02Gene08938.t1*	s24:30574668:30584952:+	88.75	8.88	109.86	0.29	No	11	Va
*PeHAK40*	*PH02Gene08939.t1*	s24:30530041:30533157:+	86.37	8.93	103.76	0.277	No	12	CM. Va
*PeHAK41*	*PH02Gene22019.t1*	s24:62929248:62935856:-	88.30	7.25	111.13	0.394	No	13	Va

MW, molecular weight; PI, isoelectric point; AI, aliphatic index; GRAVY, grand average of hydropathicity score; SP, signal peptide; TMS, transmembrane domain; PL, predicted location; CM, cell membrane; Va, vacuole.

Furthermore, the assessment of aliphatic amino acid indices revealed that the proteins in this family exhibited thermal stability ranging from 98.45 to 117.03, indicating relatively high thermal protein stability. Signal peptide analysis of the 41 members identified *PeHAK18* as the only protein with a signal peptide. All 41 proteins were found to possess predicted transmembrane segments (ranging from 9 to 14), suggesting their localization at the cytoplasmic membrane or in the vacuole. This localization pattern is consistent with the transmembrane transport function performed by HAK proteins.

### Classification and phylogenetic analysis of the *HAK* gene family

**Figure 1:**
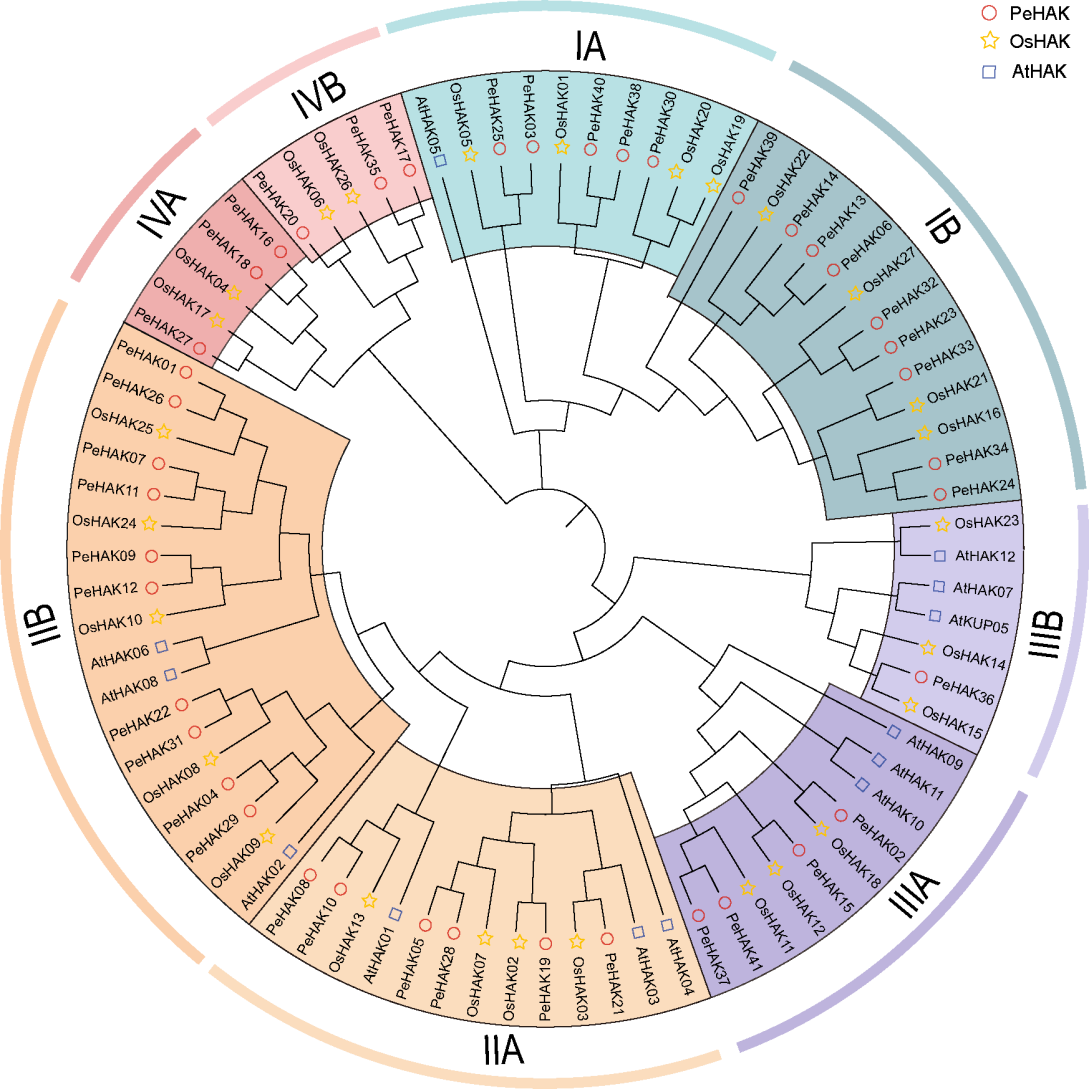
Phylogenetic analysis of *HAK* family proteins from rice (*O. sativa*), Moso bamboo (*P. edulis*), and *A. thaliana*. The *HAK* family proteins were classified into four clusters, denoted by different colors. Cluster I, II, III, and IV are represented by yellow, purple, green, and, red clusters, respectively.

Phylogenetic analysis was conducted using the full-length sequences of HAK proteins from Arabidopsis (13 proteins), rice (27 proteins), and Moso bamboo (41 proteins) (**Figure 1** **).** The analysis revealed four major clusters, each further divided into subclusters A and B. Among the *PeHAKs*, cluster I contained 14 members (IA, and IB), cluster II contained 16 members (IIA, and IIB), cluster III contained 5 members (IIIA, and IIIB), and cluster IV contained 6 members (IVA, and IVB). These members exhibited an uneven distribution between monocotyledons and dicotyledons. Notably, cluster I and cluster II had the highest representation among Moso bamboo HAK proteins, accounting for 73.17% of all *PeHAKs*.

In the IA branch of the evolutionary tree, *PeHAK40*, *PeHAK39*, *PeHAK38*, *PeHAK30*, *PeHAK3* and *PeHAK25* clustered with the identified plant high-affinity potassium transporter genes *AtHAK5*, *OsHAK01* and *OsHAK05*, and it is speculated that they may have similar functions in moso bamboo. Interestingly, Arabidopsis HAK genes did not cluster in the IV cluster, whereas four rice genes (*OsHAK04*, *OsHAK06*, *OsHAK17*, and *OsHAK26*) and six Moso bamboo genes (*PeHAK16, PeHAK17, PeHAK18, PeHAK20, PeHAK27*, and *PeHAK35*) were grouped in the IV cluster.

### Conserved structural domain, motifs, and gene structure

**Figure 2:**
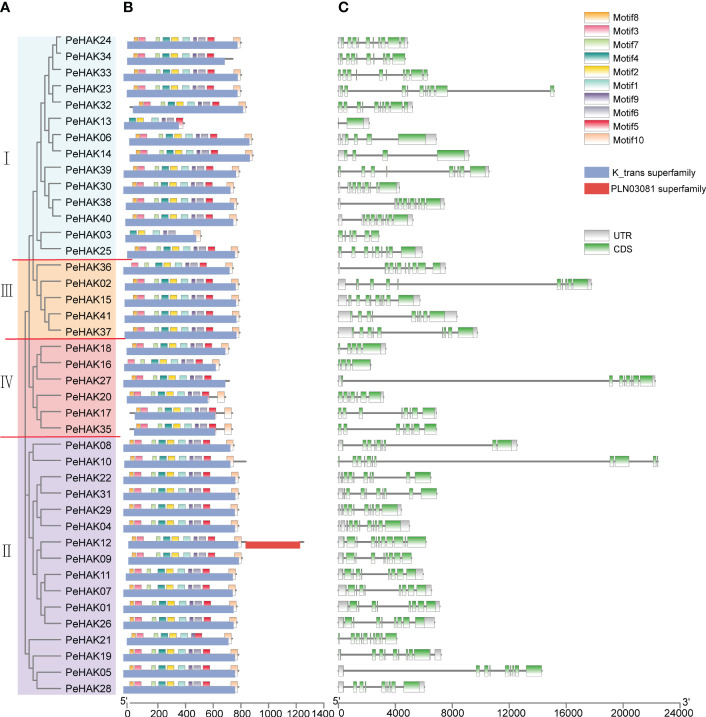
Phylogenetic relationship and sequence characteristics of PeHAK proteins. **(A)** Phylogenetic analysis of PeHAK proteins. **(B)** Conserved motifs and domains of PeHAK proteins. Conserved domains and motifs are indicated on the upper side and lower side of protein sequences, respectively. **(C)** *PeHAK* gene structures. Introns and exons are represented by black lines and green boxes, respectively.

To gain a deeper understanding of the classification and structural composition of *PeHAKs*, we analyzed their motifs, domains, and gene structures. Based on the conserved motifs and structural domains in PeHAK protein sequences, PeHAKs were classified into four clusters (**Figure 2A**), which aligned well with the results of the phylogenetic analysis. We identified ten conserved motifs in PeHAK sequences, and all members exhibited these motifs except for PeHAK34, PeHAK13, PeHAK03, PeHAK36, and PeHAK16, which exhibited partial deletions of the conserved motifs (**Figure 2B**). Additionally, we observed that all PeHAKs possess a motif known as the K_trans superfamily (**Figure 2B**), except for PeHAK12, which contains a PLN03081 superfamily motif at the C-terminal. These findings indicate a high degree of conservation in PeHAK motifs and domains. However, the gene structure of PeHAKs exhibits significant variability, with 1-10 exons and 2-10 introns (**Figure 2C**).

### Chromosomal localization and duplications of the *PeHAK* genes

**Figure 3:**
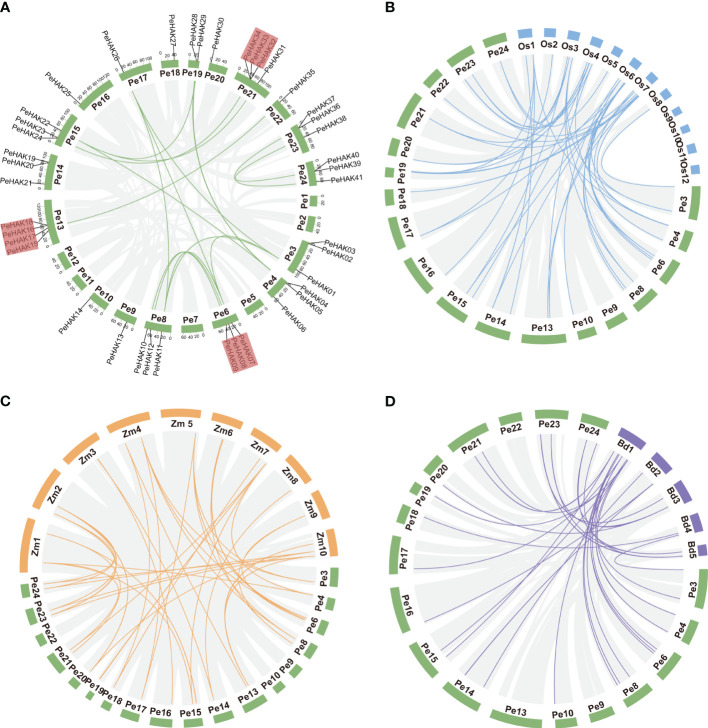
Synteny analysis of the *PeHAK* genes. **(A)** Chromosome distribution and inter-chromosomal relationships of the *PeHAK* genes. Tandem duplicated genes are set off by a red background. Scale bars represent the number of DNA bases in Mb. **(B–D)** Synteny analysis of *PeHAK* genes in *P. eduli*s and three other model plants (*O. sativa*, *Z. mays*, and *B. distachyon*). Gray lines represent aligned blocks between the paired genomes, blue, orange and purple lines indicate syntenic *HAK* gene pairs. Pe, *P. eduli*s. Os, *Oryza sativa*. Zm, *Z. mays*. Bd, **(*B*)** *distachyon*.

The distribution of *PeHAK* genes in Moso bamboo showed an uneven presence across 18 out of the 24 chromosome scaffolds. Scaffold13 and scaffold24 exhibited the highest number of occurrences, while *PeHAK* genes were absent in scaffold1, scaffold2, scaffold11, and scaffold12. Notably, scaffold13, scaffold21, and scaffold24 contained clusters of three or four genes (**Figure 3A**). Gene duplication events play a crucial role in the emergence of novel functional genes and species evolution. In this study, we employed MCScanX genomic co-linear analysis and identified 17 gene pairs resulting from fragment duplication and 22 *PeHAK* whole genome duplications (WGDs), accounting for 53.7% of the total (**Figure 3A**). Genome synteny analysis of moso bamboo and three graminaceous model plants revealed that 50, 44, and 31 moso bamboo *HAK* genes were homologous to *HAK* genes of *O. sativa*, *Z. mays*, and *B. distachyon*. Interestingly, among the three graminaceous species, homologs of HAK genes in *Z. mays* and *B. distachyon* were found in different scaffolds from moso bamboo, except for *O. sativa* chromosomes 9-12(**Figures 3B-D**).

To examine the evolution limits and the selection effects on *PeHAK* genes, we calculated Ka, Ks, and Ka/Ks values for 17 homologous *PeHAK* gene pairs (**Supplementary Table 2**). The synonymous substitution rate (Ks) represents the background base substitution rate and can be used to predict genome-wide duplication events. The Ks values of *PeHAK* gene pairs ranged from 0.0169 to 0.1111, suggesting that large-scale gene duplication events occurred as early as 54.903 million years ago and as recently as 8.0480 million years ago. Furthermore, all the Ka/Ks values for the gene pairs were below 1.0, which implies that these *HAK* genes underwent strong purifying selection during their evolution.

### Promoter characterization of *PeHAKs*

**Figure 4:**
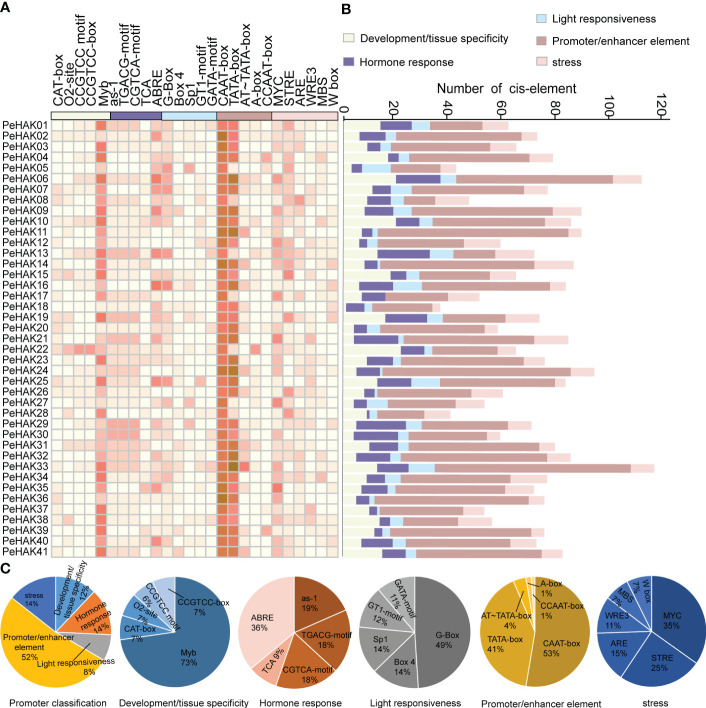
*Cis*-acting elements in *PeHAK* promoters. **(A)** The intensity of the red color indicates the number of different cis-acting elements in each *PeHAK* gene, and the five color categories on the heatmap, each representing a different functional type of cis-acting element. **(B)** The colored histograms indicate the number of different *cis*-acting elements in five categories. **(C)** The proportions of the different *cis*-acting elements in each category.

To analyze the cis-acting elements present in the 1500 bp upstream sequence of each PeHAK gene, they were categorized into five categories (**Figure 4**). The most abundant category was the promoter/enhancer element (52%), followed by stress (14%), hormone response (14%), development/tissue specificity (12%), and light responsiveness (8%) (**Figure 4A**). Within the Promoter/enhancer element category, two prevalent elements responsible for transcriptional efficiency were identified in *PeHAKs*: RNA polymerase binding sites and CAAT-boxes (53%), followed by TATA-boxes (41%). These elements play a crucial role in controlling the initiation and expression levels of *PeHAK*. Among the light responsiveness elements, the promoter G-box (49%) was widely distributed and commonly found in light-controlled genes and those regulated by environmental factors. It was followed by Box4 (14%) and Sp1 (14%). *PeHAK* promoters were found to contain hormone response *cis*-elements, including ABRE (36%), as-1 (19%), CGTCA motifs (18%), and TGACG motifs (18%), which are involved in abscisic acid (ABA), salicylic acid (SA), and methyl jasmonate (MeJA) responses, respectively (**Figures 4B, C**). Among the development/tissue specificity elements, Myb was the most abundant. Interestingly, all members of *PeHAK* contain Myb elements, suggesting that Myb is extensively involved in growth and development regulated by *PeHAK* (Dubos et al., 2010).

### Transcription profiles of *PeHAK* genes

**Figure 5:**
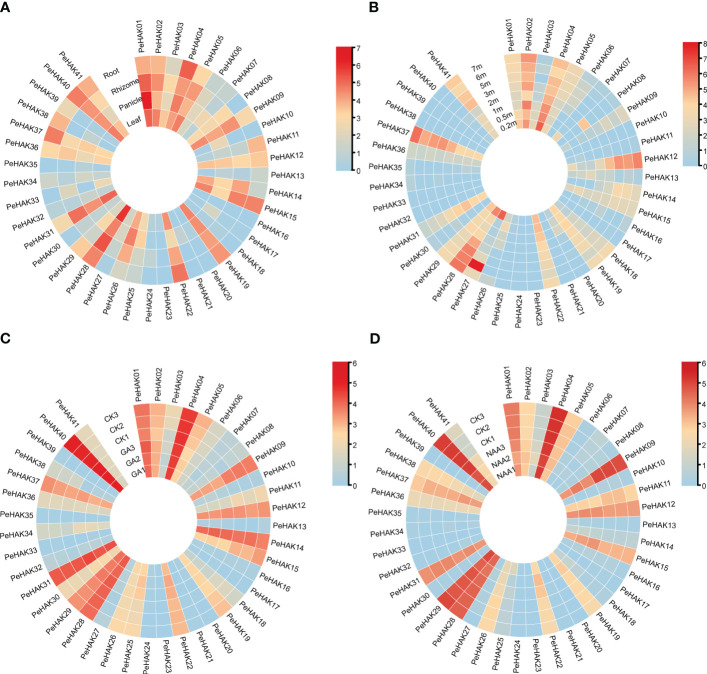
Heat map of *PeHAK* gene expression (log2^(TPM+1)^) in each tissue, and the shoots of Moso bamboo at different heights and in response to hormone treatments. **(A)** Expression in roots, rhizomes, panicles, and leaves. **(B)** Expression in young Moso bamboo shoots at different heights. **(C)** Expression under GA treatment. **(D)** Expression under NAA treatment. Relative expression levels are indicated by a color scale, with a change from blue to red indicating low to high expression.

The expression patterns of *PeHAK* genes in various tissues of Moso bamboo were investigated based on published transcriptome data. The results revealed significant differential expression of *PeHAKs* across different tissues (**Figure 5A**). More than half of the genes exhibited expression in Moso bamboo tissues. Specifically, *PeHAK01*, *PeHAK02*, *PeHAK04*, *PeHAK19*, *PeHAK28*, and *PeHAK40* remained highly expressed in different tissues. Conversely, *PeHAK15* and *PeHAK22* showed lower expression in Moso bamboo panicles but the higher expression in other tissues. *PeHAK09*, *PeHAK19*, and *PeHAK31* exhibited lower expression in roots compared to other tissues. Notably, *PeHAK04* and *PeHAK37* were highly expressed in Moso bamboo roots, suggesting their potential involvement in tissue development and nutrient uptake. Only a small proportion of *PeHAK* genes showed no expression or low expression in Moso bamboo leaves.

Further analysis of gene expression during different germination stages (**Figure 5B**) revealed dynamic expression patterns of many *PeHAK* genes. They displayed low expression in 0.2m shoots, higher expression in 0.5m shoots, and intermediate expression in 1m shoots. As the shoots grew beyond 1m, an increasing number of *PeHAK* genes exhibited high expression levels. However, *PeHAK04*, *PeHAK22*, and *PeHAK29* showed a down-regulation trend in shoots of different heights. These findings indicate that these specific *PeHAKs* are expressed during the rapid growth phase of shoots, suggesting their important roles during this period. In terms of hormone treatments, the response of PeHAK genes to gibberellic acid (GA) treatment was investigated. *PeHAK03, PeHAK04, PeHAK05, PeHAK09, PeHAK11, PeHAK14, PeHAK19*, and *PeHAK37* were down-regulated compared to the control (**Figure 5C**), indicating that their expression was inhibited by GA treatment. Conversely, *PeHAK01, PeHAK28*, and *PeHAK40* were up-regulated with GA treatment (**Figure 5C** **),** but down-regulated with naphthalene acetic acid (NAA) treatment (**Figure 5D**), suggesting individual variations in the expression of *PeHAK* genes in response to different hormones.

### The analysis of short time-series expression miner

**Figure 6:**
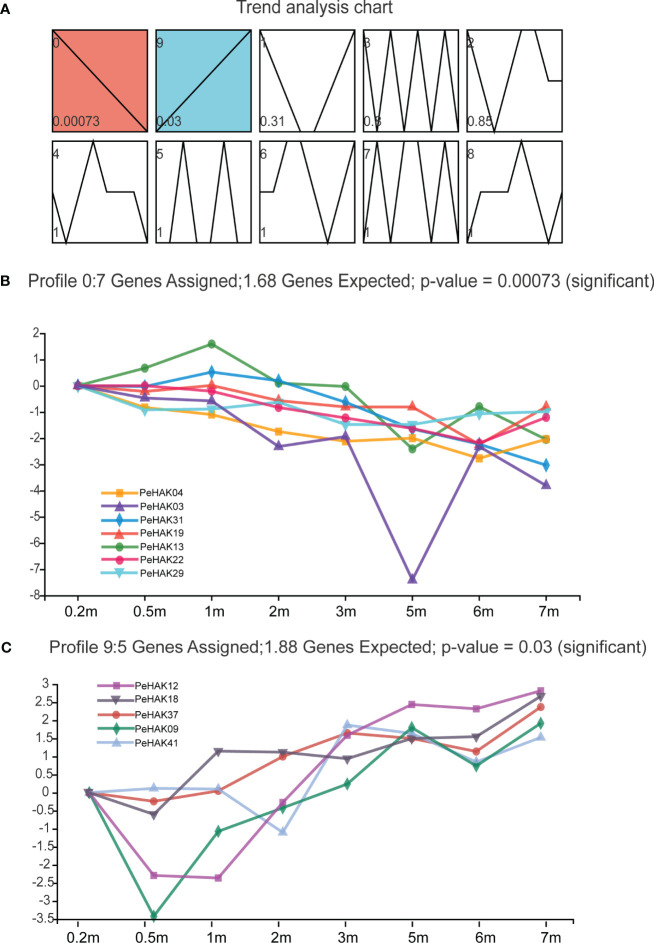
The analysis of Short Time-series Expression Miner (STEM) of *PeHAK* genes. **(A)** Trend analysis graphs produced by the STEM algorithm. Shades of blue to red color indicate different significant levels of gene expression. All 10 profiles are drawn with profile numbers denoted at the top left. The dashed line indicates the trend of expression over time, and the value in the lower-left corner is the P-value for its corresponding significance level. The right panel shows the performance of profiles 0 and profiles 9 significantly expressed genes. **(B)** Expression changes of the six genes in profile 0. **(C)** Expression changes of the six genes in profile 9.

The gene expression data was analyzed using STEM, a tool that clusters, compares, and visualizes gene expression patterns across different time points. The analysis revealed 10 distinct gene expression profiles (**Figure 6A**). Interestingly, two groups of expression profiles showed contrasting patterns. Profile 0 consisted of seven *PeHAK* genes, all of which displayed a negative correlation with shoot height (**Figure 6B**). On the other hand, profile 9 comprised five *PeHAK* genes that exhibited a positive association with the height of Moso bamboo shoots (**Figure 6C**).

### Expression patterns of *PeHAK* in response to abiotic stresses

**Figure 7:**
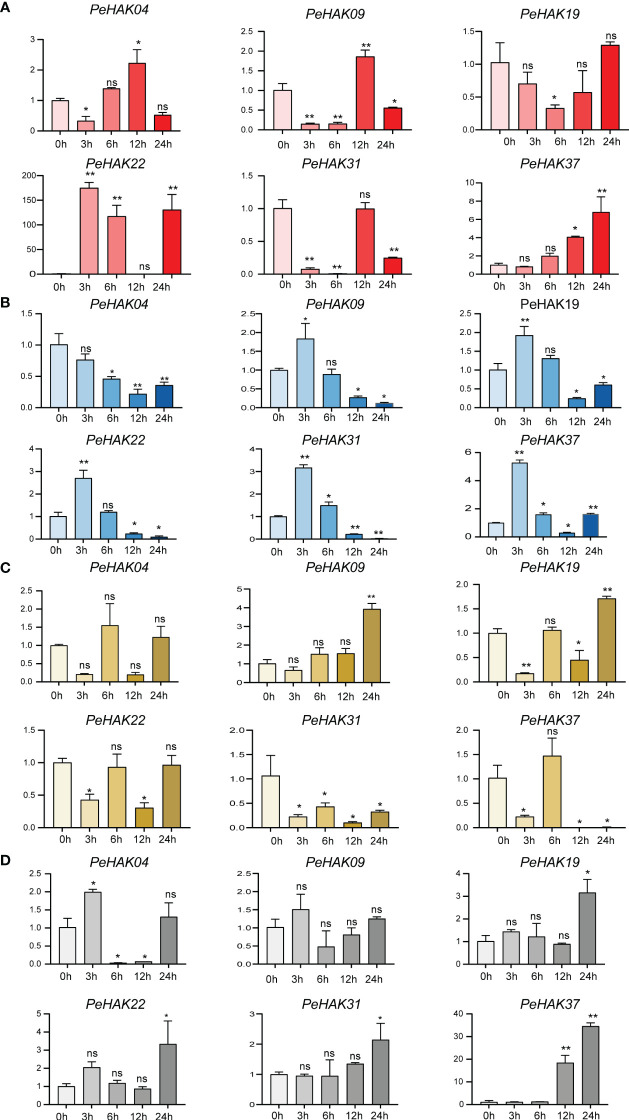
Six *PeHAK* expression profiles in Moso bamboo seedlings in response to different abiotic stress. *PeHAK* expression profiles under high temperature stress (42°C) **(A)**, under low temperature stress (4°C) **(B)**, under drought stress (30%PEG6000) **(C)**, and under 200 mM NaCl stress **(D)**. qRT-PCR was performed using three biological replicates and three technical replicates of the moso bamboo sample type. Asterisks indicate statistically significant differences between control (0h) and different treatment times (∗P ≤ 0.05, ∗∗P ≤ 0.001).

To determine the impact of different abiotic stress conditions on *PeHAK* gene expression, we investigated the effects of high temperature (42°C), low temperature (4°C), drought stress (30% PEG6000), and salt stress (200 mM NaCl) on *PeHAK* gene expression. All four abiotic stresses influenced *PeHAK* gene expression to varying extents. High-temperature treatment significantly increased the expression of *PeHAK22* and *PeHAK37* (**Figure 7A**), while it had no discernible effect on the expression of the other genes. Under low-temperature treatment, except for *PeHAK04*, the expression of *PeHAK09, PeHAK19, PeHAK22, PeHAK31*, and *PeHAK37* was down-regulated (**Figure 7B**). However, there was an initial induction of expression at 3 h and 6 h, followed by down-regulation, indicating a pattern of induction followed by inhibition. In response to drought treatment, *PeHAK09* and *PeHAK19* were up-regulated, particularly after 24 h of treatment (**Figure 7C**). On the other hand, *PeHAK31* and *PeHAK37* showed varying degrees of down-regulation in expression following drought treatment. Further analysis revealed that the expression of the other four genes was up-regulated following exposure to salt stress for 12 and 24 h (**Figure 7D**). Among them, *PeHAK37* exhibited significantly higher expression compared to the control, while *PeHAK09* showed only a slight difference in expression. *PeHAK04* demonstrated an expression pattern of induction followed by suppression. These results indicate that different stress conditions affect *PeHAK* gene expression in Moso bamboo, with each stress condition eliciting unique expression patterns for specific genes.

### GO enrichment analysis

**Figure 8:**
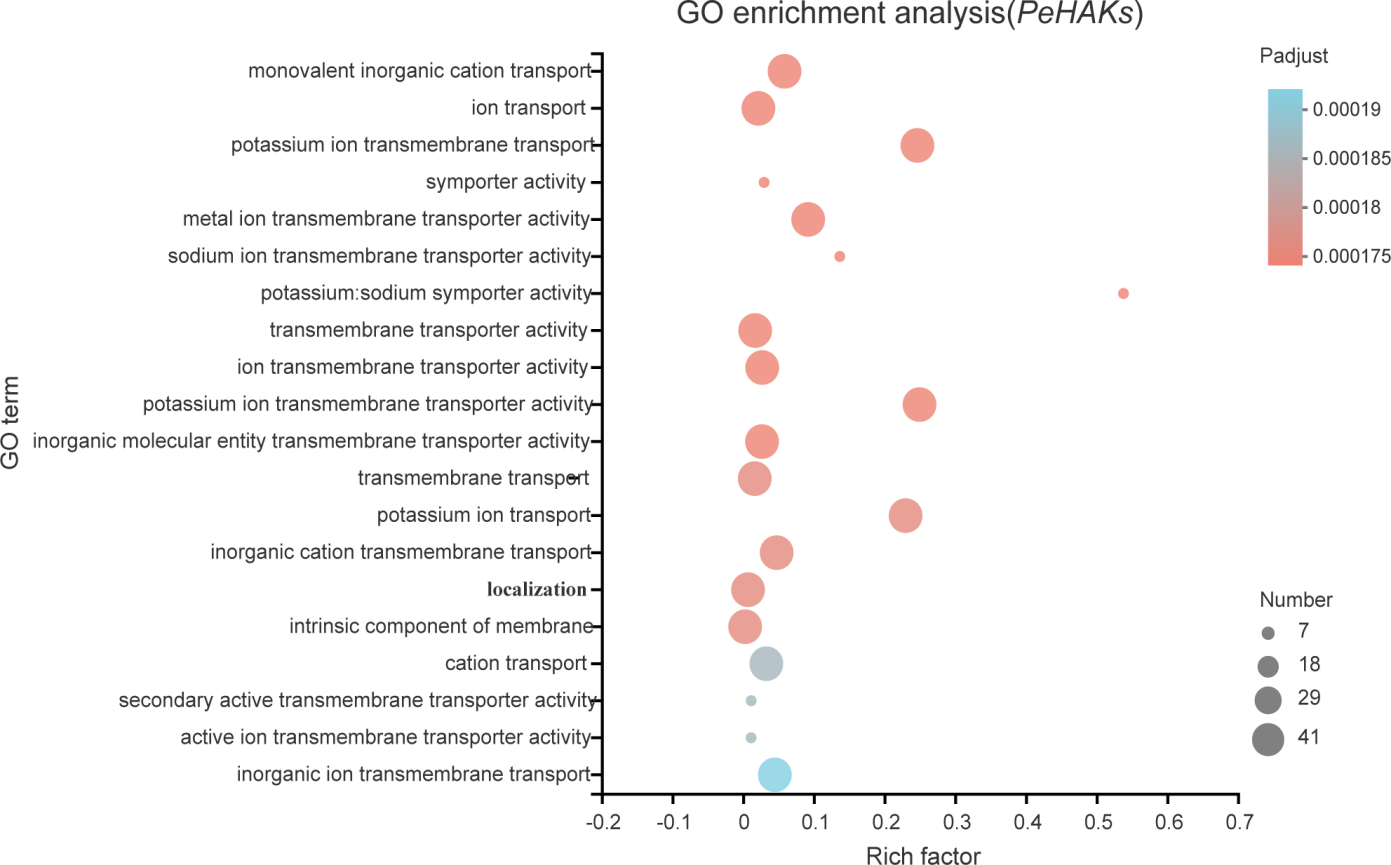
The 20 most enriched GO terms of *PeHAK* genes. The horizontal axis indicates the enrichment factor, and the size of the circle indicates the number of genes annotated with a given GO term.

To gain insights into the biological roles of the 41 *PeHAK* genes, we performed GO annotation and enrichment analysis. The top 20 GO terms are shown in **Figure 8**. The results of the GO enrichment analysis showed that *PeHAKs* are predominantly involved in potassium ion transmembrane transport, potassium ion transmembrane transporter activity, and potassium ion transport. This suggests that the main function of *PeHAK* genes is related to potassium ion transport. Additionally, we observed that the term “transport” occurs frequently in the functional module, indicating the involvement of several genes from the HAK family in ion transport. Notably, some of these genes were associated with sodium ion transporter activity, consistent with previous studies highlighting the role of *PeHAKs* in sodium ion transport (Li et al., 2018).

### Subcellular localization of PeHAK proteins

**Figure 9:**
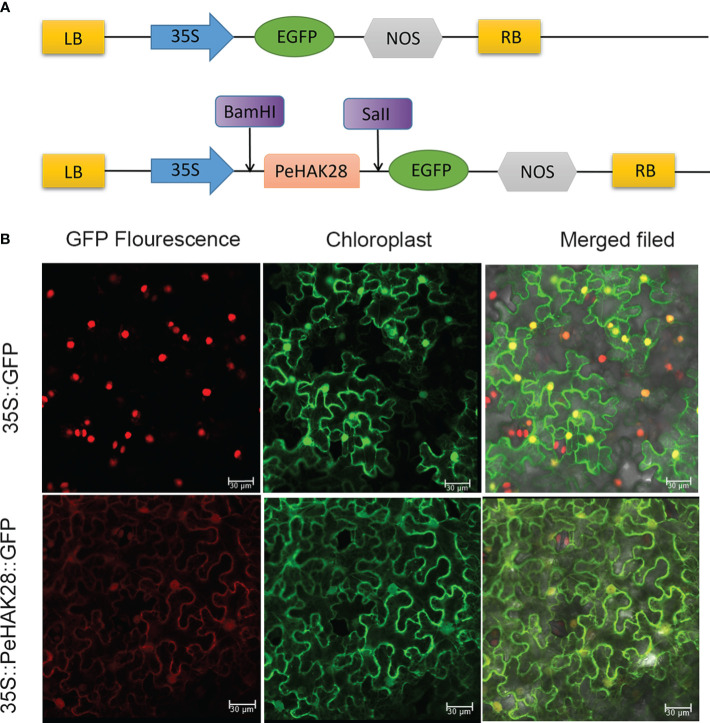
Subcellular localization of GFP-fused PeHAK28 protein. **(A)** Schematic representation of PeHAK28 vector map for subcellular localization. **(B)** Subcellular localization of PeHAK28 in tobacco cells. The fusion protein 35S-PeHAK28-GFP and the control vector were transiently expressed in tobacco leaves and then observed by fluorescence microscopy. The scale bar represents 30 μm.

To confirm the subcellular localization of PeHAK proteins in Moso bamboo, we randomly selected PeHAK28 for investigation. We constructed a recombinant expression vector, MAS-PeHAK28-GFP (**Figure 9A**). The subcellular localization of the PeHAK28 protein was determined by observing the green fluorescence signal of GFP. The results revealed that the expressed fusion protein, 35S-PeHAK28-GFP, specifically localized to the cell membrane, whereas the GFP fluorescence signal was diffuse in the control cells of the tobacco sample (**Figure 9B**). This confirms that PeHAK28 is predominantly localized in the cell membrane.

### Tissue localization analysis of *PeHAK*

**Figure 10:**
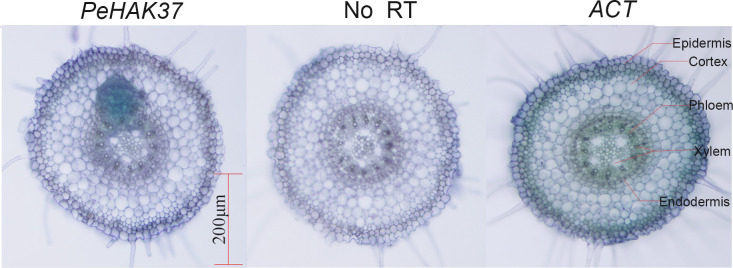
Tissue localization of *PeHAK37* using *in-situ* PCR. All samples were analyzed with BM in purple. The blue color indicates the presence of digoxigenin (DIG) labeled cDNA. *ACT* serves as a positive control, while no reverse transcription (RT) was included as a negative control. Scale bars = 200μm.

The root system plays a crucial role in potassium uptake in plants. To gain insight into the tissue localization of the *HAK* in Moso bamboo roots, we conducted an *in-situ* PCR analysis of *PeHAK37* (**Figure 10**). The aim was to investigate the specific regions within the roots where *PeHAK37* is expressed. The results shown in the figure indicate that, based on the microscopic cross-section of Moso bamboo root tips, the staining patterns of the antisense strand revealed widespread expression of *PeHAK37* in different regions of the root tips compared to the control (negative control) represented by the sense strand. The major sites of expression were observed in the lateral root primordia, while weaker expression was detected in the central cylinder and cortex.

### Validation of potassium ion transport properties

**Figure 11:**
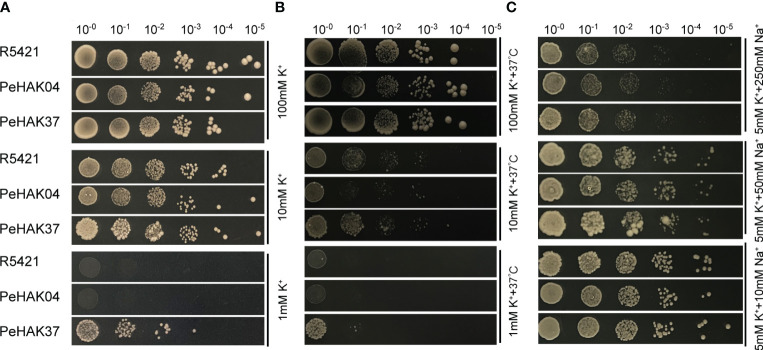
Validation of the potassium transport function of *PeHAK04* and *PeHAK37* using a yeast heterologous expression system. **(A)** Normal culture in AP medium with different K^+^ concentrations. **(B)** Incubation in AP medium containing different K^+^ concentrations at high temperature (37°C). **(C)** AP medium (5 mM K^+^) with different Na^+^ concentrations. From left to right indicates yeast transformed via a 10-fold dilution.

To validate the *HAK* transporter activity, we used the K^+^ uptake defective mutant yeast strain R5421 (MATα, Δ trk 1, trk 2:: pCK 64, his 3, leu 2, ura 3, trp 1 and ade 2) for complementary validation. The results showed little difference in growth between strain R5421 (control) transformed by the P416 vector and yeast transformed with *PeHAK04* or *PeHAK37* when grown on AP medium at 100 mM K^+^ (**Figure 11A**). In fact, the growth of the control performed slightly better than with *PeHAK04* or *PeHAK37*. When the AP medium K^+^ concentration was reduced to 10 mM, *PeHAK04* or *PeHAK37* outperformed the control for better growth compared to the control. Notably, neither the empty vector control nor *PeHAK04* grew when the concentration was reduced to 1 mM K^+^, but expression of *PeHAK37* rescued the growth defect of yeast mutant R5421, suggesting that *PeHAK37* conferred significant potassium uptake and growth in yeast at low K^+^ concentrations. In contrast, there was no significant growth difference between *PeHAK04* and *PeHAK37* at 100 mM K^+^ concentration compared to the control in AP medium at high temperature (37°C) (**Figure 11B**). However, when the concentration was reduced to 10 mM K^+^ and 1 mM K^+^, *PeHAK04* showed no significant growth difference from the control, while *PeHAK37* performed slightly better. In addition, *PeHAK37* showed better growth than the control at 37°C at high temperature and low potassium, further supporting its transporter function.

K^+^ uptake defective strain R5421 showed Na^+^ hypersensitivity, a property that can be used to determine whether exogenous genes are involved in K^+^ uptake and salt tolerance in yeast cells, and we added different concentrations of NaCl (10 mM, 50 mM, 250 mM) to 5 mM AP medium. It was found that at 10 mM Na^+^, only *PeHAK37* grew slightly better compared to the control (**Figure 11C**). However, at other concentrations, the growth of *PeHAK04*, *PeHAK37* and the control showed no significant difference and was hindered by salt stress. Further studies in other AP solid media with different Na^+^ concentrations showed no differences between *PeHAK04*, *PeHAK37* and the control. It is therefore hypothesized that *PeHAK37*-mediated K^+^ transport enhances salt tolerance in R5421 cells by a small amount.

### Analysis of protein tertiary structures

**Figure 12:**
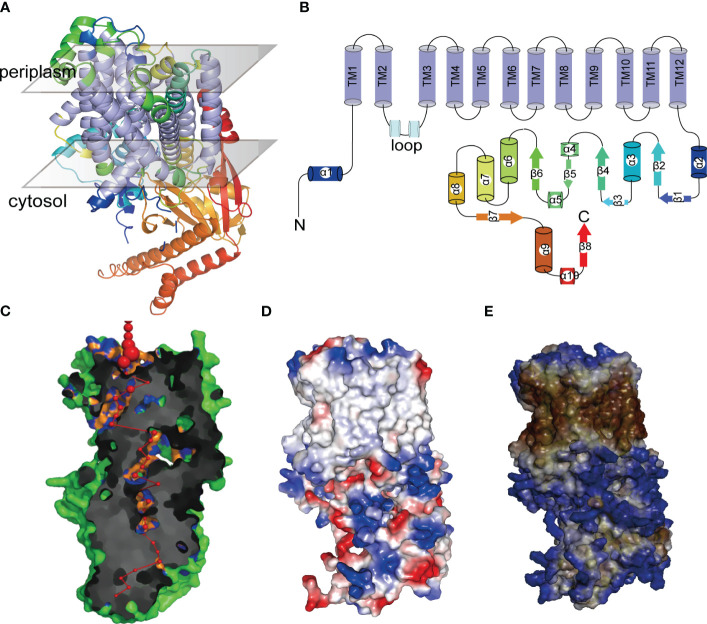
The tertiary structures of PeHAK25 protein. **(A)** Representation of PeHAK25 crystal structure. **(B)** Analysis of the distribution of transmembrane structure positions in PeHAK25 stereo structure. **(C)** Visualization of a pore section showing the pore-lining residues and the pore centers at 3A˚ steps: The red sphere represents the center of the hole at the 3A˚ step, and its size is proportional to the diameter of the hole at that point. Orange and blue represent pore-lining atoms and residues, respectively. **(D)** Mapping of the electrostatic potential onto the molecular surface. Blue and red represent positively and negatively charged regions, respectively. **(E)** Mapping of the hydrophobic group onto the molecular surface. Brown and blue represent hydrophilic and hydrophobic areas, respectively.

AlphaFold revealed that the tertiary structures of PeHAK25 protein contain a tightly arranged transmembrane structural domain consisting of 12 α-helices (**Figure 12A**). Additionally, the PeHAK25 structure has a cytoplasmic loop comprising two α-helices between the second and third transmembrane domains. The N- and C-termini are situated on the inner side of the cell membrane, where the C-terminus is longer and contains multiple α-helices and β-folds. This structure includes 12 TM residues, and the transmembrane region forms a narrow pathway through the membrane, consistent with the HAK structure (**Figure 12B**). Further prediction revealed that the pores within the TMs traverse the cell membrane (**Figure 12C**). The binding region between the cell membrane and the external environment contains several atoms and residues, with the pore center reaching its maximum size. The protein surface is hydrophobic, with the TMs serving as hydrophobic cores (**Figure 12D**). The distribution of electrostatic potential further demonstrates that the TMs have a significantly decreased electrostatic force (**Figure 12E**), with numerous charged regions at both ends of the TMs, particularly negatively charged regions towards the inner side of the cell membrane. This suggests that the distinctive structural characteristics of HAK greatly enhance the cation transport capabilities of PeHAK.

## Discussion

Several studies have shown that HAK gene family is associated with transmembrane transport of K^+^ and potassium supply in plants (Ahmad and Maathuis, 2014; Li et al., 2018). However, no study has investigated the structure and functional relationship of *HAK* in Moso bamboo. Moso bamboo is a globally cultivated species and an important crop plant known for its remarkably fast-growing shoots. Therefore, studying the molecular network and regulatory mechanisms underlying the rapid growth of bamboo shoots may provide valuable insights for cultivation and breeding (Chen et al., 2022).

In this study, several *HAK* gene family members (41) were identified in Moso bamboo compared with other plants such as rice (27), Arabidopsis (13), maize (27), tea tree (21), and wheat (56) (Ankit et al., 2022). Notably, the number of *HAK* genes is not significantly correlated with genome size (Santa-María et al., 2018). For instance, Moso bamboo has a genome size (2051 Mb) similar to its close relative maize (2066 Mb), but with a significantly higher number of *HAK* genes. Meanwhile, wheat has a genome size almost seven times that of Moso bamboo (14454 Mb) but with only 15 more *HAK* genes than Moso bamboo. This suggests that the evolution of a species is more closely linked to the number of gene families rather than the genome size. Herein, an intra-genomic covariance analysis of *PeHAK* was conducted to further understand the evolutionary patterns of *HAK* genes in Moso bamboo. Twenty pairs of duplicated *HAK* genes, including 17 tandem repeats and 3 fragment repeats, were detected in Moso bamboo genome. These repeats can promote *HAK* gene expansion and functions. Gramineae-based covariance analyses revealed a large number of homologous genes in *Z. mays*, *O. sativa*, and significantly fewer in *B. distachyon*. than in the other two species, which is consistent with previous studies in which *Z. mays*, *O. sativa* confirmed the occurrence of a genome-wide duplication event in Maso bamboo as well as a tetraploid origin (**Figure 3**). Additionally, the Ka/Ks values were consistent with previously reported large-scale replication events (7-15 million years ago) in the entire genome of Moso bamboo (Zhang et al., 2022). The Ka/Ks ratios were less than 1, indicating that purifying selection is significant in the evolution of *PeHAK* genes (Cvijovic et al., 2018). Duplication events have caused the expansion of gene family members in plants, and in addition, mutations in upstream regulatory regions and coding regions cause changes in the expression pattern and function of new members (Hashemipetroudi et al., 2023; Yaghobi and Heidari, 2023).

The evolutionary relationships among species are partially reflected in the species evolution tree (Nieves-Cordones et al., 2016). In this study, *HAK* genes from Moso bamboo, Arabidopsis, and rice were classified into four clusters (I, II, III, and IV), with HAKs present in all branches except for Arabidopsis HAKs in cluster IV where Moso bamboo had six *HAK* genes, while rice had four. This branch may be a new class of HAK members generated through gene expansion of monocot HAK family genes during the evolutionary process. Nonetheless, subsequent research should assess the function of this class of *HAK* genes. These findings indicate that the number and function of HAK gene family members are relatively conserved across different species, exhibiting similar functional characteristics.

Protein families exhibiting functional diversity among their members provide insights into the structural factors that govern protein function and their evolutionary relationships (Harms and Thornton, 2010). The crucial structural features of HAK proteins, such as the transmembrane, N-terminal, and C-terminal domains, play a significant role in determining their ability to transport and uptake K^+^ (Tascon et al., 2020). This study focused on the structural analysis of PeHAK, a membrane protein with 9-14 transmembrane structures. The pore structure, surface electrostatic potential, and surface hydrophobicity were also evaluated to understand the functional characteristics of PeHAK at the tertiary structural level. Notably, the presence of transmembrane structural domains (TMs) is crucial for predicting the functional structure of PeHAK.

Interestingly, the analysis of pore structure combined with the electrostatic potential on the protein surface revealed several negatively charged residues within the PeHAK cell membrane, which aligned with the pore structure distribution. Previous studies have indicated that the localization of charged residues plays a crucial role in transmembrane proteins (Zhou and Pang, 2018). Besides, surface electrostatic potential is closely related to ion transport rates (Busschaert et al., 2017). For instance, KUP protein (KimA) binds to negatively charged amino acid groups in the K^+^ binding site, facilitating K^+^ transport (Tascon et al., 2020). These results indicate that the transport of K^+^ in PeHAK relies on transmembrane channels formed by the membrane structural domain (TM) and the negative charge distribution on the cell membrane. However, further comprehensive studies should assess the transmembrane transport mechanism of PeHAK.

Plant growth and development are closely associated with potassium nutritional status (Amtmann et al., 2008). About 40-90% of K^+^ in plants is acquired through the root system and is transported from the subsurface to the aboveground parts via the root cycle (Lu et al., 2005). Herein, GO enrichment analysis showed that *PeHAKs* proteins are primarily associated with potassium ion transport across the cell membrane. Furthermore, an *in-situ* PCR analysis was conducted using Moso bamboo root tips to investigate *PeHAK* expression patterns (**Figure 10**). *PeHAK37* is mainly expressed in the lateral root primordia, with weaker expression in the middle column and cortex. *OsHAK5* plays a key role in K^+^ transport from roots to the above-ground parts of plants. It is highly expressed in the xylem’s thin-walled tissues and the phloem of root vascular tissues. Inactivation of *OsHAK5* leads to a reduced K^+^ concentration in xylem sap and a lower rate of K^+^ export (Li et al., 2018). Similarly, knockdown of *AtKUP7* results in decreased K^+^ uptake in the root system and reduced K^+^ concentration in the xylem sap, indicating that *AtKUP7* is involved in both the uptake and translocation of K^+^ (Han et al., 2016).This suggests that *PeHAK37* participates in long-distance K^+^ transport by utilizing the transporting tissues of the middle column, facilitated by the uptake of soil K^+^ by lateral roots. Moreover, yeast complementation experiments were performed to further confirm *PeHAK37* function in potassium ion transport. These experiments confirmed that *PeHAK37* is involved in potassium ion transport in Moso bamboo. Growth hormones influence cell expansion and division by altering ionic currents, including those of potassium (K^+^). Studies have demonstrated that HAK transporters respond to exogenous growth hormones, facilitating lateral root formation and root growth (Philippar et al., 2004; Braun et al., 2008). In addition, HAK activity is regulated by a variety of substances, including naphthalene acetic acid and gibberellins, especially for potassium signaling in low potassium environments (Santa-María et al., 2018). In Moreover, STEM expression analysis (**Figure 6**) showed different expression patterns, and it was hypothesized that these genes might play key roles in the sprouting and growth of moso bamboo and might be key regulators of the K^+^ signaling process. Overall, these results suggest that *PeHAKs*, including *PeHAK37*, are involved in potassium ion transport and growth and development of Moso bamboo, especially long-distance transport, and promotion of shoot height.

Environmental factors play a crucial role in plant growth and development (Lobell and Gourdji, 2012). Plant stress tolerance is closely associated with the expression of specific genes, such as *HAK*. *HAK* plays a key role in plant stress response (Ankit et al., 2022). For example, *HAK* genes are involved in plant response to abiotic stresses, such as drought and salt (Li et al., 2018; Yang et al., 2020). *Cis*-acting elements are DNA sequences that interact with structural genes and serve as binding sites for transcription factors. These elements regulate gene expression in plants by binding to transcription factors and controlling the timing and efficiency of gene transcription (Hernandez-Garcia and Finer, 2014). In this study, the potential role of *PeHAKs* in stress response was evaluated by examining *PeHAK* promoter regions and predicting the presence of various *cis*-acting elements associated with different abiotic stress (**Figure 4**). These elements included Myb elements (Martin and Paz-Ares, 1997; Dubos et al., 2010), a cluster of G-boxes (Menkens et al., 1995), and ABRE elements (Fujita et al., 2013) associated with hormone responses. Furthermore, qRT-PCR analysis was performed under high temperature, low temperature, drought, and salt stresses to determine *PeHAK* expression patterns (**Figure 7**). Most HAK gene family members of the Mao bamboo responded to at least two abiotic stresses, indicating that HAK genes regulate responses to environmental stress.

Furthermore, HAK gene family members participate in the crosstalk between hormone signals, such as ABA and IAA (Yang et al., 2020; Cai et al., 2021). Herein, transcriptome data showed that *PeHAK* was up-regulated under NAA and GA treatments compared with the controls (**Figure 5**). However, some members, including *PeHAK01, PeHAK04, PeHAK09, PeHAK11, PeHAK38*, and *PeHAK40*, were down-regulated in NAA treatment, while *PeHAK03, PeHAK05, PeHAK09*, and *PeHAK15* were down-regulated in GA treatment (**Supplementary Table 3**). Previous studies have found that HAK is involved in potassium transport in response to various signaling stimuli, such as the ABA signaling pathway under drought stress (Li et al., 2018). In addition, several transcription factors were identified, including RAP2.11 (related to AP2.11), bHLH121 (basic helix-loop-helix121), and others that bind to the HAK promoter in response to the absence of K^+^ and activate the expression of HAK, and the overexpression of these transcription factors can increase root growth in the absence of K^+^ (Kim et al., 2012; Santa-María et al., 2018). These findings suggest that the external environment stimulates certain *PeHAKs*, thus enhancing potassium content and Moso bamboo resistance. In summary, *PeHAK* genes play a crucial role in plant stress response to abiotic stresses and hormone crosstalk. Notably, *PeHAK* genes enhance the resistance of Moso bamboo to environmental stresses by modulating potassium content.

## Conclusion

This is the first study related to genome-wide identification and comprehensive analysis of *HAK* genes in Moso bamboo. In this study, the expression profiles of *PeHAKs* and their role in the rapid growth of bamboo shoots were evaluated. In addition, the role of *HAK* in potassium ion transport was evaluated based on yeast mutant experiments. Moreover, the underlying mechanism of transmembrane transport was analyzed by studying the spatial structure of the protein. Therefore, this research provides valuable insights into the evolution and functions of *PeHAKs* in the development of various plant organs. *PeHAKs* are promising candidate genes for further exploration and innovation in transgenic breeding programs involving *graminaceous* plants.

## Data availability statement

The original contributions presented in the study are included in the article/**Supplementary Material**. Further inquiries can be directed to the corresponding author.

## Author contributions

HG: Writing – review & editing, Writing – original draft, Formal analysis, Data curation, Conceptualization. JT: Writing – review & editing, Software, Investigation, Formal analysis, Data curation. YJ: Writing – original draft, Software, Methodology. BH: Writing – original draft, Software, Investigation, Formal analysis, Data curation. RM: Writing – original draft, Validation, Software, Methodology. MR: Writing – review & editing. GQ: Writing – review & editing, Visualization, Validation, Software, Methodology. ZZ: Writing – review & editing, Writing – original draft, Validation, Funding acquisition, Data curation.

## Supplementary material

Supplementary Table 1 Cis-element analysis of *PeHAK* genes.

Supplementary Table 2 Ka_Ks analysis of gene pairs in *PeHAK* gene duplicated events.

Supplementary Table 3 Expression analysis of the *PeHAK* genes.

Supplementary Table 4 All primers were used in this study.
